# Impact of pharmacy worker training and deployment on access to essential medicines for children under five in Malawi: a cluster quasi-experimental evaluation

**DOI:** 10.1186/s12913-017-2530-7

**Published:** 2017-09-11

**Authors:** Joseph B. Babigumira, Solomon J. Lubinga, Alisa M. Jenny, Erin Larsen-Cooper, Jessica Crawford, Charles Matemba, Andy Stergachis

**Affiliations:** 1 Global Medicines Program, Departments of Global Health and Pharmacy, University of Washington, Harris Hydraulics Building 1510 San Juan Road, Box 357965, Seattle, WA 98195 USA; 2 Pharmaceutical Outcomes Research and Policy Program, Department of Pharmacy, University of Washington, Seattle, WA USA; 3 VillageReach, Seattle, WA USA; 4 VillageReach, Lilongwe, Malawi

**Keywords:** Health workforce, Supply chain, Quasi-experiment, Impact evaluation, Malawi, Essential medicines, Malaria, Pneumonia, Diarrhea

## Abstract

**Background:**

Poor access to essential medicines is common in many low- and middle-income countries, partly due to an insufficient and inadequately trained workforce to manage the medicines supply chain. We conducted a prospective impact evaluation of the training and deployment of pharmacy assistants (PAs) to rural health centers in Malawi.

**Methods:**

A quasi-experimental design was used to compare access to medicines in two districts where newly trained PAs were deployed to health centers (intervention) and two districts with no trained PAs at health centers (comparison). A baseline household survey and two annual post-intervention household surveys were conducted. We studied children under five years with a history of fever, cough and difficulty in breathing, and diarrhea in the previous two weeks. We collected data on access to antimalarials, antibiotics and oral rehydration salts (ORS) during the childrens’ symptomatic periods. We used difference-in-differences regression models to estimate the impact of PA training and deployment on access to medicines.

**Results:**

We included 3974 children across the three rounds of annual surveys: 1840 (46%) in the districts with PAs deployed at health centers and 2096 (53%) in districts with no PAs deployed at health centers. Approximately 80% of children had a fever, nearly 30% had a cough, and 43% had diarrhea in the previous two weeks. In the first year of the program, the presence of a PA led to a significant 74% increase in the odds of access to any antimalarial, and a significant 49% increase in the odds of access to artemisinin combination therapies. This effect was restricted to the first year post-intervention. There was no effect of presence of a PA on access to antibiotics or ORS.

**Conclusion:**

The training and deployment of pharmacy assistants to rural health centers in Malawi increased access to antimalarial medications over the first year, but the effect was attenuated over the second year. Pharmacy assistants training and deployment demonstrated no impact on access to antibiotics for pneumonia or oral rehydration salts for diarrhea.

## Background

The UN Sustainable Development Goals called for improved access to safe, effective, quality and affordable essential medicines and vaccines for all [1]. However, access to the most life-saving medicines and medical supplies in low- and middle-income countries (LMICs) remains unacceptably low. For example, it is estimated that only 46% of adults and 49% of children living with HIV in LMICs had access to treatment with antiretroviral medications at the end of 2015 [2]. The public sector availability of essential medicines in 23 LMICs in 2012 was estimated at only a mean value of 40% [3]. In sub-Saharan Africa, children bear the brunt of the adverse morbidity and mortality impact of lack of access to essential medicines, particularly as it applies to medicines for the treatment of malaria, pneumonia and diarrhea [4, 5].

The reasons for persistently low access to essential medicines in LMICs include inadequate financing, regulatory issues, lengthy procurement processes, poor logistics management, and a lack of qualified health workers to manage the medicines supply chain [6–9]. Malawi reflects many of these constraints — poor health systems infrastructure and working conditions [10]; an ongoing health worker crisis [11]; and limitations in product availability, supply chain knowledge, and transportation [12]. Additionally, the 2011–2013 Malawi Health Sector Strategic Plan identified only five pharmacists in the country’s public health system and estimated that only 24% of the established positions for pharmacy technicians were filled [13]. And despite substantial improvements between 1990 and 2013, Malawi still has one of the highest under-five mortality rates in the world [14].

Task-shifting, the delegation of tasks from more- to less-sophisticated health workers, is the reality in sub-Saharan Africa, driven by severe health workforce shortages. Task-shifting has the potential to increase the effective health workforce and to be cost-effective [15, 16]. In Malawi, the logistics management and supply chain functions of health center pharmacies rely on task-shifting from pharmacists and pharmacy technicians to clinical health workers such as medical assistants and nursing aides [17, 18], and, in our experience, even to grounds laborers and security guards. The lack of training in medicines logistics management of these lower-level cadres leads to inefficiencies in supply chain management and clinical care, which may lead to shortages in essential medicines resulting in adverse health consequences for patients [11, 17, 19].

To address the shortage of well-trained pharmacy workers, VillageReach, a Seattle-based international non-governmental organization with expertise in supply chain management, initiated an innovative pharmacy assistant (PA) training program, in conjunction with the Malawi College of Health Sciences (MCHS) and the Malawi Ministry of Health. The training program, initiated in 2013, provides two years of training towards a Certificate in Pharmacy, i.e., pharmacy assistant. Malawi previously trained and deployed pharmacy assistants at district and central hospitals, but the program was discontinued nearly two decades ago, resulting in a shortage of pharmacy workers at the primary level [13, 20]. The primary level of health service delivery in Malawi is the frontline of a three-tier health system, operating at the village level. It consists of village clinics, health posts, dispensaries, maternities, health centers and community and rural hospitals. The secondary level operates at the district level and the tertiary level operates nationally.

There is limited evidence on the impact of strengthening human resources capacity for medicines supply on access to medicines and on health outcomes [18]. To bridge this knowledge gap, we designed a prospective evaluation of the impact of pharmacy worker training and deployment on access to essential medicines for malaria, pneumonia, and diarrhea, i.e., artemisinin-based combination therapies (ACTs), antibiotics, and oral rehydration salts (ORS), respectively, the leading causes of childhood mortality in Malawi [5]. The pre-specified hypothesis was that pharmacy assistant training and deployment, as implemented by VillageReach and partners, would improve access to these essential medicines through improved management, logistics information flow for essential medicines, and supply chain functions at the primary health centers.

## Methods

### Study design

**Fig. 1:**
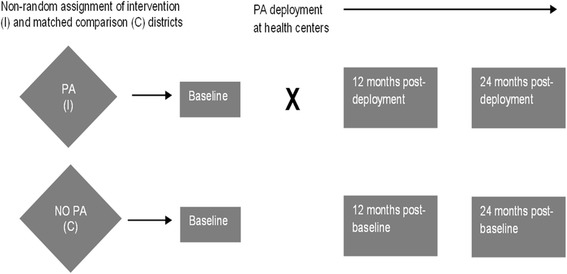
Quasi-experimental design of the impact evaluation of pharmacy assistant training and deployment in Malawi. PA—pharmacy assistant; I—Intervention; C—comparison; X—timing of intervention. Adapted from Lubinga et al. [22]

We used a quasi-experimental design (Fig. 1), with data derived from three population-based cluster household surveys: one pre-implementation sample conducted in 2014 and two post-implementation samples conducted in 2015 and 2016. The primary outcome as measured in the survey was access to ACTs, antibiotics, and ORS. Access to medicines was defined as the percentage of children with non-severe malaria, pneumonia, or diarrhea in the last two weeks who obtained a full course of ACT, antibiotics, or ORS, respectively. This definition was adopted from the Malawi Demographic and Health Survey (DHS), 2010 [21]. The details of the study design have been reported elsewhere [22] and are summarized below.

### Study setting

The study was conducted in four districts of Malawi: Ntchisi and Machinga (intervention) and Dedza and Chikwawa (comparison). The comparison districts were matched on region of the country; presence of high burden of malaria, pneumonia, and diarrhea; access to basic services; geographic location; and socioeconomic status of residents. Within each intervention district, a single health center was the site of PA student deployment: Khuwi health center in Ntchisi and Ntaja health center in Machinga. The health centers of Dolo in Chikwawa and Lobi in Dedza were chosen as comparison sites.

### The intervention

The intervention was a newly developed and implemented pharmacy assistant training program. Briefly, the training program is composed of both didactic and an extended practicum training [23]. During the first year, students underwent ten weeks of class-based instruction at the MCHS. After this period, half of the students were deployed for a five-month period of field training supervised by a pharmacy technician at a district hospital, while the other half underwent additional didactic training. The students then switched places for the next five months, i.e., the students who underwent field training undergo didactic training, and vice versa. During the second year, half the students were deployed to rural health centers for five months while the other half underwent additional didactic training, before switching places. Therefore, during the second year, student trainees were present at participating rural health centers for ten of twelve months. The potential benefits of the practicum were two-fold: PA students trained in an environment that is characteristic of their future workplace, and PA students filled an immediate human resources gap at the primary health center.

### Study procedures

Following a baseline household survey conducted in March 2014, we conducted annual household surveys in March 2015 and March 2016. A two-stage cluster sampling design was used. In the first stage, enumeration areas (EAs) close to intervention and comparison health centers were identified. In the second stage, households were randomly selected within EAs by identifying a central location and going from house-to-house guided by a random number generator.

We identified households, using self-report by parent or caregiver, with at least one child under five years of age with symptoms of non-severe malaria, pneumonia, or diarrhea in the previous two weeks (i.e., fever; cough, difficulty breathing or a rapid respiratory rate; or diarrhea, respectively). Eligible children were included upon consent from the parent or caregiver and excluded if they had severe disease. The questionnaire, adapted from the 2010 Malawi Demographic and Health Survey and Women’s questionnaires [21], included details about household characteristics, care-seeking behavior, and medicine-use. The questionnaire was translated into Chichewa and back-translated into English to confirm reliability and validity of the survey instrument. The questionnaires were piloted and refined by local field teams for clarity and ease of understanding among the target population. Individuals who conducted data collection underwent two-day trainings before each round of data collection.

### Sample size and power

The study was powered to detect increases in access to ACTs, antibiotics, and ORS of 50%, 40% and 80% respectively, with 80% power and 5% precision. Given the prevalence of fever, pneumonia symptoms, and diarrhea of 35%, 7%, and 18%, respectively [21], and assuming 1.5 children per household, a non-response rate of 10%, and a design effect of 1.5 to account for within-cluster homogeneity, in the primary outcome [21, 24], we estimated needing to sample 4150 households, divided equally between intervention and comparison sites, to enroll the required number of children with the symptoms of interest.

### Statistical analyses

Data from the questionnaires were double-entered and cross-validated using the Census and Survey Processing System, CSPro (US Census Bureau, USA). All analyses were conducted using R.

Methods described by the Malawi DHS were used to calculate household wealth index scores from data on ownership of goods and housing characteristics [21]. Participant characteristics and the overall illness distribution were summarized over the three independent annual samples.

Using a logit link function, three empiric specifications of the mean were estimated:

1. All post-intervention data for 2015 and 2016 were grouped together and the simple difference-in-differences effect was estimated. The coefficient is the difference in the difference in log-odds of access to medicines in the pre-intervention versus the post-intervention periods, when comparing the intervention group with the comparison group.
2. Dummy variables were created for the year. The coefficient is the difference in the difference in log-odds of access to medicine in the pre-intervention versus the post-intervention periods, when comparing the intervention group with the comparison group, assuming the intervention effect is the same in both post-intervention periods (2015 and 2016).
3. Dummy variables for year were interacted with the intervention indicator, permitting the estimation of differential effects (trends) by year. The coefficient is the difference in the difference in log-odds of access to medicine in the pre-intervention versus the first year (2015) post-intervention and the difference in the pre-intervention versus the second year (2016) post-intervention when comparing the intervention group with the comparison group.

All models were estimated using maximum likelihood, and standard errors were adjusted for heteroscedasticity and clustering at enumeration area level and family level. In all cases unadjusted and adjusted models were estimated. Models were adjusted for child’s age and sex, care giver’s age and sex, level of education and marital status, and the household socioeconomic status (wealth index).

## Results

### Participant demographic and clinical characteristics

We included 3974 children in the study across the three rounds of annual surveys: 1840 (46%) in the districts to which PAs were deployed (intervention) and 2096 (53%) in the districts to which no PAs were deployed (comparison). The demographic and clinical characteristics of the children and their caregivers are summarized in Table 1. The average age of children in the study was two years, and approximately half of study children were male. Caregivers were, on average, 29 years old, predominantly female (98%), and a majority had some primary education. Half the families were in the middle of five quantiles of socioeconomic status. Nearly 80% of children included in the study were reported to have had a fever, nearly 30% were reported to have had a cough, and 43% were reported to have had diarrhea in the previous two weeks.

**Table 1:** Demographic and clinical characteristics

	Intervention (*n* = 2096, 52.74%)	Comparison (*n* = 1840, 46.3%)	Total (*n* = 3947)
Age of child, years, mean (SD)	2.34 (1.3)	2.15 (1.3)	2.25 (1.3)
Male child, n (%)	843 (48.9%)	991 (50.7%)	1834 (49.9%)
Number of persons in household, mean (SD)	5.39 (1.8)	5.24 (1.8)	5.32 (1.8)
Age of primary caregiver, mean (SD)	29.11 (7.9)	29.15 (8.6)	29.13 (8.3)
Female primary caregiver, n (%)	1765 (97.8%)	2008 (98.7%)	3810 (98.3%)
Education level of primary caregiver			
None, n (%)	348 (20.2%)	187 (9.6%)	535 (14.6%)
Primary, n (%)	1263 (73.5%)	1487 (76.4%)	2750 (75.1%)
Above primary, n (%)	106 (6.1%)	270 (13.8%)	376 (10.2%)
Socioeconomic status quantile			
Highest, n (%)	-	-	-
Second Highest, n (%)	421 (24.4%)	292 (14.9%)	713 (19.4%)
Middle, n (%)	927 (53.8%)	884 (45.2%)	1811 (49.2%)
Second Lowest, n (%)	324 (18.8%)	561 (28.7%)	885 (24.0%)
Lowest, n (%)	49 (2.8%)	217 (11.1%)	266 (7.2%)
Child’s symptoms in last two weeks			
Fever, n (%)	1390 (76.3%)	1676 (80.8%)	3096 (78.7%)
Cough, n (%)	515 (28.3%)	578 (27.8%)	1101 (28.0%)
Diarrhea, n (%)	783 (43.0%)	898 (43.3%)	1697 (43.2%)

### Impact of pharmacy assistant training and deployment

**Fig. 2:**
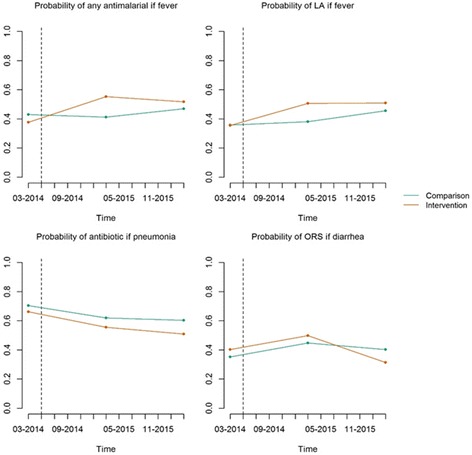
Impact of pharmacy assistant training and deployment. The dotted line represents the timing of the intervention

The results of having a trained PA at health centers on access to medicines are shown in Table 2 and Fig. 2. With regard to the impact of PA training and deployment at health centers on access to any antimalarial, model 1 shows a significant effect of the program on the odds of access to any antimalarial. This effect is maintained after adjusting for year fixed effects (model 2). However, model 3 shows that the effect of PA training and deployment on access to any antimalarial was restricted to the first year post intervention.

**Table 2:** Regression analyses

	Any antimalarial						AL						Antibiotic						ORS					
	Model 1		Model 2		Model 3		Model 1		Model 2		Model 3		Model 1		Model 2		Model 3		Model 1		Model 2		Model 3	
	Unadj	Adj	Unadj	Adj	Unadj	Adj	Unadj	Adj	Unadj	Adj	Unadj	Adj	Unadj	Adj	Unadj	Adj	Unadj	Adj	Unadj	Adj	Unadj	Adj	Unadj	Adj
Constant	−0.285^**^	−0.624^**^	−0.285^**^	−0.625^**^	−0.285^**^	−0.611^**^	−0.587^***^	−1.097^***^	−0.587^***^	−1.100^***^	−0.587^***^	−1.088^***^	0.869^***^	0.951^**^	0.869^***^	0.947^**^	0.869^***^	0.949^**^	−0.613^***^	−0.975^**^	−0.613^***^	−0.929^**^	−0.613^***^	−0.925^**^
	(0.116)	(0.255)	(0.116)	(0.254)	(0.116)	(0.253)	(0.092)	(0.247)	(0.092)	(0.244)	(0.092)	(0.244)	(0.160)	(0.445)	(0.160)	(0.446)	(0.160)	(0.446)	(0.175)	(0.417)	(0.175)	(0.424)	(0.175)	(0.424)
Intervention	−0.222	−0.188	−0.222	−0.187	−0.222	−0.188	−0.010	0.026	−0.010	0.029	−0.010	0.028	−0.199	−0.513^**^	−0.199	−0.513^**^	−0.199	−0.513^**^	0.208	0.121	0.208	0.116	0.208	0.113
	(0.180)	(0.169)	(0.180)	(0.169)	(0.180)	(0.169)	(0.170)	(0.163)	(0.170)	(0.163)	(0.170)	(0.163)	(0.234)	(0.245)	(0.234)	(0.245)	(0.234)	(0.245)	(0.261)	(0.268)	(0.261)	(0.268)	(0.261)	(0.269)
Post Intervention	0.045	0.019					0.252^*^	0.230^*^					−0.420^**^	−0.453^**^					0.315	0.339				
	(0.156)	(0.156)					(0.133)	(0.138)					(0.211)	(0.213)					(0.219)	(0.230)				
Intervention* Post-Intervention	0.590^**^	0.565^**^	0.588^**^	0.560^**^			0.374^*^	0.335	0.364	0.323			−0.123	−0.078	−0.133	−0.087			−0.339	−0.297	−0.298	−0.251		
	(0.234)	(0.224)	(0.235)	(0.225)			(0.222)	(0.216)	(0.223)	(0.217)			(0.306)	(0.307)	(0.308)	(0.308)			(0.298)	(0.302)	(0.305)	(0.309)		
year 2015			0.026	−0.014	−0.085	−0.123			0.172	0.132	0.082	0.042			−0.329	−0.362	−0.379	−0.395			0.543^**^	0.582^**^	0.392	0.422^*^
			(0.176)	(0.177)	(0.206)	(0.209)			(0.146)	(0.152)	(0.166)	(0.173)			(0.249)	(0.246)	(0.270)	(0.274)			(0.228)	(0.236)	(0.243)	(0.253)
year 2016			0.062	0.051	0.169	0.157			0.328^**^	0.324^**^	0.413^**^	0.410^**^			−0.488^**^	−0.524^**^	−0.451^*^	−0.500^**^			0.046	0.051	0.227	0.251
			(0.164)	(0.163)	(0.176)	(0.175)			(0.151)	(0.155)	(0.170)	(0.174)			(0.221)	(0.227)	(0.233)	(0.233)			(0.244)	(0.254)	(0.253)	(0.267)
year 2015* Intervention					0.794^***^	0.762^***^					0.529^**^	0.488^*^					−0.054	−0.036					−0.002	0.070
					(0.282)	(0.281)					(0.251)	(0.252)					(0.361)	(0.375)					(0.350)	(0.353)
year 2016* Intervention					0.409	0.384					0.223	0.179					−0.197	−0.130					−0.612^*^	−0.597^*^
					(0.264)	(0.251)					(0.262)	(0.253)					(0.374)	(0.359)					(0.332)	(0.341)
N	2873	2873	2873	2873	2873	2873	2873	2873	2873	2873	2873	2873	1036	1036	1036	1036	1036	1036	1552	1552	1552	1552	1552	1552
Log-Likelihood	−1963	−1922	−1963	−1921	−1961	−1919	−1939	−1893	−1938	−1891	−1937	−1889	−679	−653	−678	−652	−678	−652	−1041	−1022	−1033	−1014	−1031	−1010
AIC	3935	3871	3937	3873	3935	3871	3887	3813	3886	3811	3885	3810	1365	1333	1366	1335	1368	1337	2090	2072	2077	2057	2073	2053

*AL* Artemether Lumefantrine, *ORS* Oral Rehydration Salts, *Unadj* unadjusted models, *Adj* models adjusted for child’s age and sex, care giver’s age and sex, level of education and marital status, and the household socioeconomic status (wealth index)

^*^ *p* < 0.1; ^**^ *p* < 0.05; ^***^ *p* < 0.01

The results of the impact of PA training and deployment on access specifically to ACTs mirror those of the impact of having trained PAs at health centers on access to any antimalarial. Model 1 shows a significant effect of the program on the odds of access to Artemether-Llumefantrine, which is maintained after adjustment for year fixed effects (model 2), and restricted to the first year post-intervention (model 3). The analysis showed no effect of PA training and deployment on access to antibiotics or ORS (Table 2 and Fig. 2).

## Discussion

During the study, we tracked access to tracer essential medicines at intervention and comparison primary health centers to assess possible differential access to medicines. In this three-year prospective quasi-experimental impact evaluation of pharmacy assistant training and deployment to rural health centers in Malawi, we found mixed results: an increase in access to antimalarials over the first year that was attenuated during the second year. Additionally, there was no impact on access to antibiotics for pneumonia and oral rehydration salts for diarrhea. Therefore, our pre-specified hypothesis held for antimalarials over the first year but did not hold at all for antibiotics and ORS.

The differential impact of PA training and deployment may be explained in part by the difference in funding structure and supply chain dynamics for malaria, antibiotics, and ORS in Malawi during the study period [25]. Antimalarials are frequently donor-supported and channeled to tertiary health facilities through an informed “push” system that, in turn, distribute to lower level health facilities. Other essential medicines including antibiotics and ORS are more often supplied through the government central medical stores with limited donor support and channeled through a traditional “pull” system. Donor-supported commodities are also subject to interruptions in supply that are not necessarily amenable to improvements with interventions at the various levels of health centers. However, interventions at tertiary health centers are more likely to increase access to the donor-supported antimalarials than access to other essential medicines [25]. While the reasons for the attenuation in the effect of pharmacy worker training and deployment on access to antimalarials in the second year were unclear, it is not uncommon for low-income countries such as Malawi to experience supply chain shocks leading to interruptions in supply of medicines, including donor-funded or donor-supplied medicines [26].

Previous studies have evaluated the impact of health worker training on knowledge and clinical practice behavior in LMICs and reported mixed results, with didactic training appearing to be less successful than interactive and in-practice training [27]. The PA training model included both didactic and in-practice training, although the outcome of interest was access to tracer commodities and not clinical practice behavior. Impact evaluations of task-shifting have also been performed and generally demonstrate that health workers with less training are successful at performing a variety of clinical tasks [28–33]. However, we found no prospective evaluations of the impact of training and deploying a new cadre of health workers that are trained at a level comparable to the PAs.

Pharmacy workers are critical to health service delivery in LMICs, particularly in rural areas, where medicines are the mainstay of primary health care. The VillageReach PA training program expanded the health workforce in Malawi and the deployment of PAs appeared to increase community access to antimalarials in the short-term. In addition, monitoring data collected by VillageReach during supervision visits to students showed that PA students led to increased data quality (from 55 to 73% report accuracy) and improved adherence to storeroom management (from 72 to 79% adherence) and dispensing standards (from 41 to 60% adherence) (Jessica Crawford, personal communication). However, the intervention neither had sustained impact on access to antimalarials over two years, nor had impact on access to other medicines evaluated in this study.

Other possible reasons for our mixed results include the fact that poor access to medicines in LMICs is a multi-faceted problem with multiple contributing factors over-and-beyond the lack of sufficient and well-trained personnel. Other contributing factors include: poor infrastructure and bottlenecks at higher levels of the health system including poor financing, regulatory barriers, long procurement process, mismanagement, and corruption [6–9, 34]. It is unlikely that training and deploying PAs alone would have been enough to increase community access to medicines within the study timeframe without concurrent interventions also targeting the other factors that limit access to essential medicines.

In Malawi, healthcare in general and essential medicines in particular are intended to be free-of-charge, but patients often have to pay out-of-pocket in private pharmacies and drug shops due to frequent stock-outs [34]. Therefore, care seeking often involves multiple facilities, usually starting with the government facility, and progressing to the private sector — private clinics, drug shops, non-drug-shop retail outlets — in search of medicines. In navigating this system of facilities, patients may have sought essential medicines outside of our radius of PA deployment and impact evaluation. This would make it difficult to demonstrate an impact of PA training and deployment on access to medicines within the immediate vicinity of health facilities.

The impact evaluation was conducted over a relatively short time period given the effort to demonstrate change at the population level and was conducted with students (rather than graduated and deployed PAs). We would expect that the bulk of the benefits of this ongoing PA training program would accrue in the future as the newly trained PAs complete the learning curve and establish themselves in their practice settings. In addition, it is anticipated that the facility level improvements observed in the logistics management and information system reporting and ordering of medicines will, overtime, improve forecasting and supply planning at higher levels. As such, this study may have underestimated the impact of PA training and deployment.

Training programs are likely to succeed if there is ready employment for the trained individuals, and the trained individuals are retained in the health care system. In Malawi, there are challenges in recruitment of health workers, with complex recruitment processes and a poorly-funded recruitment agency within the Ministry of Health, leading to a health worker vacancy rate of over 50% in the public sector [35]. Retention is also a major problem because health workers in Malawi work in challenging environments and are poorly remunerated and can be under-motivated [36, 37]. Retention of trained health workers requires the deployment of remuneration and non-remuneration strategies such as provision of housing, improved practice conditions, and opportunities for career advancement and further training [38, 39]. While the long term success of the PA training program will depend on such efforts, it is important to note that at the time of the conclusion of this impact evaluation, 100% of the graduates of the PA training program were employed by the Ministry of Health.

One limitation of this study is that we were unable to randomize PA deployment to intervention and comparison sites due to practical limitations, such as logistical constraints and political influences [22]. This means that, despite the use of the differences-in-differences method, the observed effect of PA training and deployment, or lack thereof, may be explained in part by differences in baseline characteristics between the intervention and control groups. Additionally, we are only able to assess the combined impact of training and deployment and are unable to differentiate between the two [22]. Another limitation that could have influenced the results is that we used a single pre-intervention survey and two post-intervention surveys. As such, we are unable to establish a pre-intervention trend in access to medicines. This means that we had to make an assumption that the pre-intervention one-time access estimate amounted to a trend in access. Given the timing of the decision to perform the evaluation, it was the best possible design despite this limitation. The lack of two pre-intervention time points also means that a selection threat remains, and precludes our ability to test empirically whether this threat was present, or not, in the evaluation. Finally, the outcomes in this study were based exclusively on data from household surveys. This precluded a more holistic view of the impact of pharmacy worker training and deployment on access to medicines by excluding system-level effects. The overall project included health-facility-based experiential and time-motion surveys and data from these studies will be reported separately.

## Conclusions

Pharmacy assistant training and deployment to rural health centers in Malawi increased access to antimalarial medications for children under five years in the short-term but this effect was attenuated over the subsequent year. The intervention had no effect on access to antibiotics for pneumonia and oral rehydration salts for diarrhea. While pharmacy assistants are viewed as valuable additions to the health workforce in Malawi and will likely contribute to long-term systemic benefits, their short-term impact on access to essential medicines was limited. This study points to the challenges of expanding the health workforce in low- and middle-income countries in general and illustrates the importance of performing impact evaluations of health workforce interventions despite difficulties in isolating the effects of one intervention from other complex health system issues.

## Abbreviations

ACTs: Artemisinin-Based Combination Therapies
DHS: Demographic and Health Surveys
EA: Enumeration Area
LMICs: Low- and Middle-Income Countries
MCHS: Malawi College of Health Sciences
ORS: Oral Rehydration Salts
PA: Pharmacy Assistant
